# Multi-omics profiling of oral microbial functional signatures and systemic immune–metabolic features in perinatal depression

**DOI:** 10.1016/j.bbih.2026.101300

**Published:** 2026-07-13

**Authors:** Kexin Xue, Chunfei Hu, Zhehao Lin, Xiaokai Mao, Haihua Zhu, Yanyi Xie, Qiong Luo, Fudong Zhu

**Affiliations:** a Stomatology Hospital, School of Stomatology, Zhejiang University School of Medicine, Zhejiang Provincial Clinical Research Center for Oral Diseases, Key Laboratory of Oral Biomedical Research of Zhejiang Province, Cancer Center of Zhejiang University, Engineering Research Center of Oral Biomaterials and Devices of Zhejiang Province, Hangzhou, Zhejiang, China; b Department of Stomatology, The Fourth Affiliated Hospital, Zhejiang University School of Medicine, Yiwu, Zhejiang, China; c Department of Obstetrics and Gynecology, Shaoxing Maternity and Child Health Care Hospital, Shaoxing, Zhejiang, China; d College of Computer Science and Technology, Zhejiang University, Hangzhou, Zhejiang, China; e Department of Obstetrics, Women’s Hospital, Zhejiang University School of Medicine, Hangzhou, Zhejiang, China

**Keywords:** Perinatal depression, Oral microbiome, Immune–metabolic features, Multi-omics integration, Lipopolysaccharide, Bile acids

## Abstract

**Background:**

Perinatal depression (PND) occurs during a period marked by profound endocrine, metabolic, and immune adaptation. Although alterations in immune–metabolic regulation have been reported in PND, how such changes manifest across distinct biological compartments remains unclear. The oral mucosal ecosystem represents an immunologically active interface with direct connections to systemic circulation, yet its functional characteristics in PND have been insufficiently explored. In this study, we examined whether PND is characterized by differences in oral microbial functional profiles alongside systemic immune–metabolic features.

**Methods:**

We performed an integrated multi-omics analysis combining salivary shotgun metagenomics and untargeted serum metabolomics in 31 women with PND and 32 healthy controls. Oral microbial taxonomic composition and inferred functional profiles were analyzed together with circulating metabolites related to endocrine and immune processes. Cross-omics analyses were used to evaluate overall concordance as well as pathway- and feature-level associations between microbial functional signals and host metabolic features.

**Findings:**

The oral microbiome of women with PND showed largely preserved community structure and diversity, while differences were observed at the level of inferred functional pathways, including enrichment of lipopolysaccharide biosynthesis and virulence-associated functional categories. Concurrently, the serum metabolome exhibited differences in steroid-related metabolites, bile acid profiles, and lipid mediator–associated features involved in immune modulation, including putatively annotated resolvin D5. Global concordance between oral microbial functional profiles and systemic metabolomic patterns was limited; however, reproducible associations were observed at the pathway and feature levels, such as an inverse association between the relative abundance of the genus *Abiotrophia* and the bile acid taurochenodeoxycholate-7-sulfate.

**Interpretation:**

Together, these findings describe concurrent differences in oral microbial functional signatures and systemic immune–metabolic features in women with PND, occurring in the context of minimal changes in microbial community composition. The limited global concordance and selective pathway-level correspondence across omic layers are consistent with asynchronous patterns of biological variation during the perinatal period. These observations support the potential relevance of the oral–systemic axis as a non-invasive perspective for characterizing biological heterogeneity associated with perinatal depression.

## Highlights

• Perinatal depression is associated with differences in systemic immune–metabolic features during the perinatal period.
• Salivary metagenomic profiling shows largely preserved oral microbial community structure alongside differences in inferred functional profiles.
• Serum metabolomic profiles display pathway-level variation involving bile acid, steroid-related, and lipid mediator–associated metabolic classes.
• Cross-omic analyses reveal limited global correspondence with selective associations between oral microbial functional features and systemic metabolism.
• Oral microbial functional profiling offers a non-invasive perspective for characterizing immune–metabolic variation associated with perinatal depression.

## Graphical abstract

**Figure:**
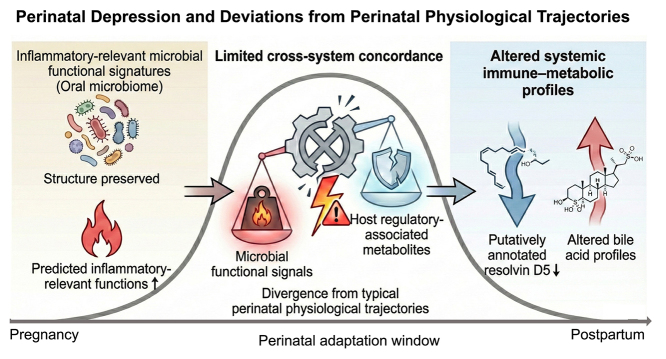

## 1 Introduction

Perinatal depression (PND) affects approximately 10%–20% of mothers worldwide and has been linked to a range of adverse outcomes for both mothers and offspring, including preterm birth, altered neurodevelopment, and longer-term emotional and cognitive difficulties (Mitchell et al., 2023, World Health Organization, 2024, Jarde et al., 2016, Rogers et al., 2020). Beyond its psychiatric manifestations, pregnancy and the postpartum period represent a biologically dynamic phase characterized by substantial endocrine, metabolic, and immune adaptation required to support maternal and fetal homeostasis (Mor and Cardenas, 2010, Howard and Khalifeh, 2020). Increasing evidence suggests that PND is accompanied by deviations from typical perinatal physiological trajectories across multiple biological systems, rather than reflecting dysfunction within a single pathway (Patterson et al., 2024). Nevertheless, clinical identification of PND continues to rely largely on self-report instruments, underscoring the limited integration of biological information into current assessment frameworks and motivating interest in system-level biological characterization (Levis et al., 2020, Accortt et al., 2023).

Bidirectional interactions between the microbiota and the central nervous system are closely intertwined with host immune and metabolic regulation (Cryan et al., 2019, Margolis et al., 2021). While the majority of existing work has focused on the gut microbiome, the oral cavity constitutes a distinct and immunologically active microbial ecosystem that interfaces directly with host barrier tissues and systemic circulation (Lamont et al., 2018, Hajishengallis and Chavakis, 2021). Recent studies have reported associations between oral microbial features and depressive or anxiety-related symptoms, with functional signals implicating pathways related to lipopolysaccharide biosynthesis, virulence-associated functional categories, and microbial energy metabolism (Malan-Müller et al., 2024). Importantly, such functional differences have been observed in the absence of pronounced shifts in overall microbial community structure, suggesting that functional profiling may capture biologically relevant variation not reflected by taxonomic composition alone. This distinction may be particularly relevant during the perinatal period, when endocrine and immune transitions are pronounced, and host sensitivity to microbial-derived signals may be altered (Zhu et al., 2025, Park and Im, 2025).

At the systemic level, perinatal metabolic regulation undergoes marked reorganization. Steroid-related metabolites, bile acids, and lipid mediators play central roles in coordinating immune modulation and metabolic homeostasis, and variation in these pathways has been implicated in mood disorders and stress-related conditions (Li et al., 2022, Fleishman and Kumar, 2024). However, oral microbial functional characteristics and circulating metabolic features are rarely examined jointly within the same individuals, limiting insight into how compartment-specific biological variation may relate across biological systems in the context of PND.

To address this gap, we performed an integrated multi-omics analysis combining salivary shotgun metagenomics and untargeted serum metabolomics in women with PND and healthy controls. This approach enables characterization of oral microbial functional signatures alongside systemic immune–metabolic features. By focusing on a sensitive perinatal window, this study seeks to describe potentially asynchronous system-level patterns of biological variation associated with PND and to examine the potential relevance of saliva-based microbial functional profiling as a non-invasive perspective that may capture distinct dimensions of immune–metabolic variation in perinatal mental health.

## 2 Materials and methods

### 2.1 Study design and participant recruitment

This cross-sectional study was conducted at the Women’s Hospital, Zhejiang University, under Institutional Review Board approval (IRB-20230240-R). Participants were recruited during late pregnancy or the early postpartum period, a time window characterized by dynamic physiological changes. Diagnosis followed a two-step protocol, including initial screening using the Edinburgh Postnatal Depression Scale (EPDS) (Cox et al., 1987, Levis et al., 2020), followed by diagnostic confirmation with the Mini International Neuropsychiatric Interview (M.I.N.I.) (Sheehan et al., 1998).

Women in the PND group had EPDS scores ≥13 and met criteria for a current major depressive episode, whereas healthy controls were required to have EPDS scores < 7. Individuals with intermediate EPDS scores (7–12) were excluded to minimize diagnostic ambiguity. Additional exclusion criteria included antibiotic use within the preceding three months (Dethlefsen and Relman, 2011) and the presence of chronic systemic inflammatory conditions, in order to reduce potential confounding effects on microbial and metabolic profiles.

**Fig. 1:**
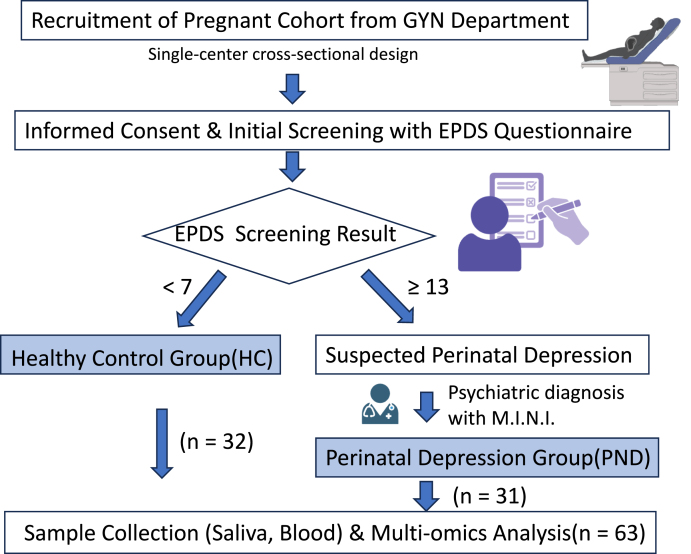
Study design and participant recruitment workflow. Participants were recruited from the obstetrics and gynecology department and screened using the Edinburgh Postnatal Depression Scale (EPDS). Women with EPDS scores ≥ 13 received diagnostic evaluation using the Mini International Neuropsychiatric Interview (M.I.N.I.) and were assigned to the perinatal depression group (PND). In contrast, those scoring < 7 were assigned to the healthy control group (HC). Saliva and blood samples were collected during late pregnancy or the early postpartum period for multi-omics analyses (n=63).

Detailed cohort characteristics and sampling distribution are provided in Appendix A. The overall workflow for participant recruitment, screening, diagnostic assessment, and biological sample collection is summarized in Fig. 1.

### 2.2 Sample collection and processing

Unstimulated whole saliva samples were collected in the morning prior to food intake, beverage consumption, or oral hygiene procedures in order to reduce short-term behavioral and dietary influences on oral microbial profiles (Navazesh, 1993). Samples were immediately placed on ice following collection and transferred to −80 °C storage until DNA extraction to preserve microbial DNA integrity (Armstrong et al., 2021).

Total microbial DNA was extracted using standardized protocols following established procedures. Shotgun metagenomic sequencing was performed to enable high-resolution characterization of both taxonomic composition and inferred functional profiles of the oral microbiome. Raw sequencing reads underwent quality control steps, including removal of low-quality reads, adapter trimming, and filtering of host-derived DNA contamination, using established pipelines (Chen et al., 2018, Salter et al., 2014).

### 2.3 Metagenomic processing and functional annotation

High-quality metagenomic reads were processed for taxonomic and functional profiling using a standardized workflow (Beghini et al., 2021, Franzosa et al., 2018). Raw paired-end reads were quality-filtered using Fastp (v0.23.0), with a base quality cutoff of Q20. Host-derived reads were removed before downstream analysis. High-quality non-host reads were assembled de novo using MEGAHIT (v1.2.9), retaining contigs with a minimum length of 300 bp. Open reading frames were predicted from assembled contigs using Prodigal (v2.6.3). Gene sequences with lengths ≥100 bp were retained and translated into amino acid sequences for downstream annotation analyses. Redundant genes were clustered using CD-HIT (v4.6.1) at 90% sequence identity and 90% coverage to generate a non-redundant gene catalog. Clean reads were mapped back to the non-redundant gene catalog using SOAPaligner (soap2.21 release), with an insert size range of 300 to 500 bp and a mapping identity threshold of 95%, for gene abundance estimation. Taxonomic annotation was performed using DIAMOND (v2.0.13; blastp; E-value ≤ 1e−05) against the NR database (release 20230830). Functional annotation was conducted using DIAMOND (v2.0.13; blastp; E-value ≤ 1e−05) against the Kyoto Encyclopedia of Genes and Genomes (KEGG) database (release 20230830) to summarize microbial metabolic pathway representation based on annotated gene content (Kanehisa and Goto, 2000). To complement general metabolic pathway profiling, sequences were additionally annotated against the Virulence Factor Database (VFDB; release 20240301) (Liu et al., 2019) in order to quantify aggregated categories of virulence-associated functional potential commonly encoded in oral microbial genomes. This analysis was intended to capture functional dimensions related to microbial adhesion, secretion, and related processes, rather than to infer active pathogenicity, infection, or host inflammatory responses. Virulence-associated features were therefore analyzed at the level of grouped functional categories, independent of overall taxonomic composition.

Functional abundance tables were normalized by gene length and sequencing depth prior to downstream statistical analyses, following established recommendations for metagenomic compositional data (Weiss et al., 2017).

### 2.4 Serum metabolomics analysis

Peripheral venous blood samples were collected after an overnight fast (≥8 h) to reduce short-term dietary influences on serum metabolomic profiles during the perinatal period. Serum was separated by centrifugation and stored at −80 °C until analysis to preserve metabolic stability (Yin et al., 2015).

Untargeted metabolomic profiling was performed using liquid chromatography coupled with mass spectrometry (Want et al., 2013). Metabolomic data preprocessing was performed using a standardized analytical workflow. Features with more than 20% missing values within any group were excluded. Remaining missing values were imputed using the minimum value across all samples. Data were normalized by sum normalization, and features with QC relative standard deviation greater than 30% were filtered out. Log10 transformation was applied before downstream analyses.

Principal component analysis and multivariate statistical analyses were conducted using the ropls R package (v1.6.2). For PLS-DA, Pareto scaling was applied, and permutation testing was performed with 200 permutations. Metabolite annotation and pathway classification were performed with reference to the KEGG COMPOUND and KEGG Pathway databases (kegg_v20230830). HMDB was also used as a reference database for metabolite annotation. KEGG pathway enrichment analysis was performed using scipy in Python (v1.0.0), with p<0.05 considered statistically significant. Heatmap visualization, clustering analysis, and correlation analysis were also implemented using scipy in Python (v1.0.0).

Metabolite annotations were assigned based on accurate mass, retention time, and MS/MS spectral similarity, corresponding primarily to MSI confidence levels 2–3. Accordingly, reported metabolite identities represent putative annotations rather than definitive structural confirmation. Downstream analyses emphasized pathway- and class-level patterns relevant to endocrine- and immune-related metabolic processes, including steroid-related metabolites, bile acids, amino acid and redox pathways, and lipid mediator-associated features. These analyses were intended to summarize coordinated metabolic variation rather than to infer specific biochemical mechanisms or causal relationships.

### 2.5 Multi-omics integration and statistical analysis

Integrated multi-omics analyses were designed to describe molecular variation within each omics layer and to evaluate patterns of correspondence between oral microbial functional profiles and systemic metabolic features (Argelaguet et al., 2018, Singh et al., 2019). Analyses proceeded in two stages: within-omics characterization to summarize group-associated patterns of variation, followed by cross-omics assessments to examine global concordance and pathway-level correspondence across data layers.

For clinical and demographic variables, continuous variables were compared using the Welch t-test and categorical variables using Fisher’s exact test. Alpha diversity of the salivary microbiome was assessed using the Chao1 and Shannon indices. Beta diversity was calculated using Bray–Curtis dissimilarity and visualized by principal coordinates analysis. Group differences in alpha diversity were evaluated using the Wilcoxon rank-sum test. Education level, smoking history, and alcohol intake were collected as categorical self-report variables. As a supplementary exploratory analysis, these variables were examined according to their original categorical coding using db-RDA to assess their potential associations with microbiome variation.

Within each omics layer, exploratory multivariate analyses were used to summarize overall data structure, and feature-level statistical testing was performed to identify group-associated differences after appropriate normalization and multiple-testing correction (Weiss et al., 2017, Benjamini and Hochberg, 1995). For serum metabolomics, PCA and PLS-DA were performed using the ropls R package (v1.6.2). For PLS-DA, Pareto scaling was applied, and model evaluation was performed using 200 permutations. Pathway enrichment, clustering, heatmap generation, and correlation analyses were implemented using scipy in Python (v1.0.0). Multiple-testing correction was performed using the Benjamini–Hochberg false discovery rate procedure where applicable. These analyses were intended to assess whether the serum metabolome exhibited coordinated variation in endocrine- and immune-related metabolic pathways and whether the oral microbiome showed differences in inferred functional pathway representation, rather than to infer specific biological mechanisms.

To evaluate cross-omic relationships, global structural similarity between salivary metagenomic functional profiles and serum metabolomic profiles was assessed using Procrustes analysis of distance matrices (González-Domínguez et al., 2024). In parallel, Mantel tests were applied to examine associations between subsets of microbial functional features and metabolite modules (Anderson, 2006). These approaches were used to assess patterns of concordance and correspondence and were not intended to infer causal or directional relationships between microbial and metabolic features.

Feature-level associations were further explored using Spearman correlation analyses with multiple-testing correction, focusing on aggregated and biologically interpretable functional categories. Given the compositional nature of metagenomic functional profiles, correlation analyses were interpreted as exploratory and were used to identify reproducible patterns rather than definitive feature-level relationships. Accordingly, emphasis was placed on pathway-level functional patterns and cross-omic consistency rather than on individual correlations.

As a secondary analysis, supervised machine-learning models were constructed to assess whether selected microbial and metabolic features contained internal discriminative information between women with perinatal depression and healthy controls (Vabalas et al., 2019). Model training and evaluation were conducted using nested cross-validation to minimize overfitting, with feature selection and parameter tuning restricted to training folds (Krstajic et al., 2014, Cawley and Talbot, 2010). Classification performance was interpreted as an estimate of internal separability rather than as a predictive model for clinical deployment.

### 2.6 Software and reproducibility

All analyses were conducted using established bioinformatics and statistical software environments, following standard practices for multi-omics data analysis (Tierney, 2012). Analytical workflows and parameter settings were based on previously validated pipelines and applied consistently across samples. To improve transparency and reproducibility, the main analytical workflow, software versions, database releases, and key processing parameters used in this study are reported in the Methods above. Statistical analyses and figure preparation were performed on macOS.

## 3 Results

### 3.1 Participant characteristics

A total of 63 participants were included in the final analysis, including 31 women with perinatal depression (PND) and 32 healthy controls (HC). Key demographic and clinical characteristics of the two groups are presented in Table 1. The PND and HC groups were comparable with respect to maternal age, pre-pregnancy body mass index, gestational or postpartum timing at sampling, parity, and mode of delivery, with no statistically significant differences observed for these variables (all p>0.05). In contrast, EPDS scores were markedly higher in the PND group than in the HC group (p<0.001), consistent with group definitions based on the study design (Cox et al., 1987, Levis et al., 2020).

**Table 1:** Participant characteristics at enrollment and sampling.

Variable	PND (n = 31)	Control (n = 32)	p-value
Age, years	29.42 ± 5.55	30.66 ± 3.49	0.296
Gestational week at enrollment	38.32 ± 1.54	38.72 ± 1.08	0.243
Prepregnancy BMI, kg/m^2^	22.46 ± 5.65	21.57 ± 2.43	0.426
Gestational weight gain, kg	12.83 ± 5.30	13.55 ± 4.02	0.546
EPDS score	14.90 ± 2.94	1.31 ± 1.94	<0.001

Late pregnancy at sampling, n (%)	21 (67.7%)	23 (71.9%)	0.788
Married, n (%)${}^{a}$	28 (90.3%)	28 (87.5%)	1.000
Education level (≥ college), n (%)${}^{a}$	15 (48.4%)	23 (71.9%)	0.057
Household income, n (%)${}^{b}$	2 (6.5%)	4 (12.5%)	0.672
Any smoking, n (%)${}^{a}$	9 (29.0%)	5 (15.6%)	0.237
Any alcohol use, n (%)${}^{a}$	14 (45.2%)	7 (21.9%)	0.064

Data are mean ± SD or n (%). P values for continuous variables from the Welch t-test. P values for categorical variables from the Fisher exact test. ${}^{a}$ Denominators reflect non-missing responses within each group (PND n=31, Control n=32). ${}^{b}$ Proportion of participants with household income above the sample median category. ${}^{c}$ Sampling period classification followed the definitions described in the Methods (late pregnancy vs. early postpartum).

### 3.2 Serum metabolomic profiles reveal systematic but partial group separation

**Fig. 2:**
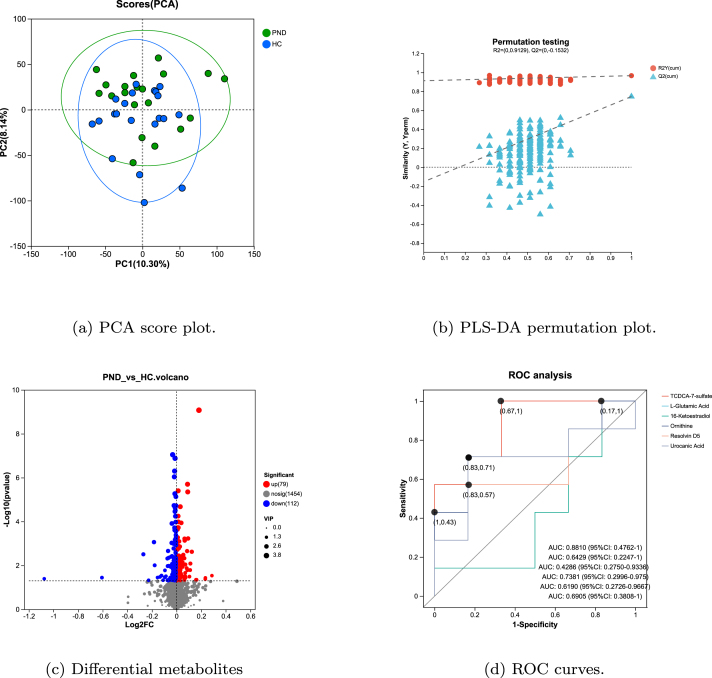
Serum metabolomic profiles in women with perinatal depression (PND) and healthy controls (HC). (a) PCA showed the overall metabolomic structure with partial overlap between groups. (b) PLS-DA suggested partial group separation, and permutation testing indicated limited model predictability. (c) Differential metabolite analysis identified a subset of metabolites with significant between-group differences. (d) ROC analysis showed modest discriminatory performance for the selected metabolite panel and individual metabolites.

Untargeted serum metabolomic profiling was conducted to examine systemic metabolic variation between women with perinatal depression (PND) and healthy controls (HC). At the global level, principal component analysis (PCA) showed substantial overlap between PND and HC samples, indicating limited separation in overall metabolomic structure (Fig. 2a) (Brunius et al., 2016). Partial least squares discriminant analysis (PLS-DA) revealed a degree of separation along selected components (Fig. 2b) (Wold et al., 2001). Permutation testing suggested that the supervised model captured non-random group-related structure within the data (p<0.05) (Forsgren et al., 2025).

Feature-level differential analysis identified a subset of metabolite features that differed between groups after false discovery rate correction, with both higher and lower relative abundances observed in the PND group (Fig. 2c). Examples of metabolites contributing to these differences included bile acid derivatives such as *taurochenodeoxycholate-7-sulfate*, amino acid–related metabolites such as *urocanic acid*, steroid-related intermediates, and lipid mediator–associated features including putatively annotated *resolvin D5* (Serhan, 2014).

Pathway enrichment analysis indicated that group-associated metabolite features were preferentially represented in pathways related to bile acid metabolism, amino acid and redox metabolism, and steroid hormone biosynthesis (Fig. 2d) (Kanehisa and Goto, 2000, Wishart et al., 2018). Taken together, these results describe coordinated pathway-level variation in the serum metabolome, occurring in the context of limited global separation between groups.

### 3.3 Oral microbiome shows preserved global structure with differences in inferred functional profiles

**Fig. 3:**
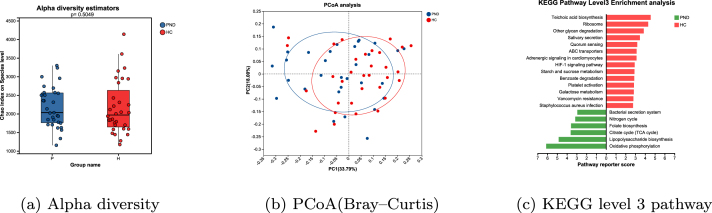

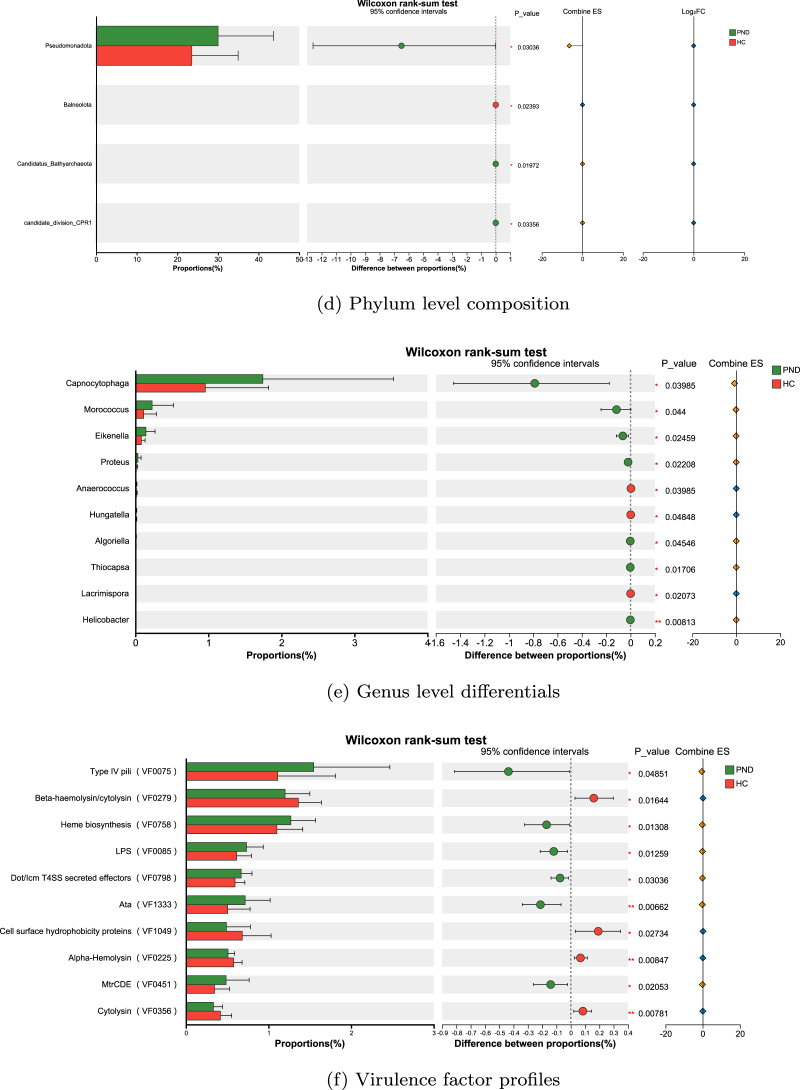
Salivary metagenomic diversity and functional profiles in women with perinatal depression (PND) and healthy controls (HC). (a) Alpha diversity, assessed by the Chao1 and Shannon indices, was comparable between groups. (b) Beta diversity based on Bray–Curtis dissimilarity showed substantial overlap between groups at the global community level. (c) KEGG level 3 pathway analysis indicated functional differences between groups despite the largely preserved global community structure. Taxonomic and virulence characteristics of the salivary microbiome in women with perinatal depression (PND) and healthy controls (HC). (d) Phylum-level composition was broadly similar between groups, consistent with preserved global community structure. (e) Genus-level differential analysis identified a limited set of taxa with significant between-group differences. (f) Virulence factor profiling showed differences in selected virulence-associated functional categories between groups.

**Fig. S1:**
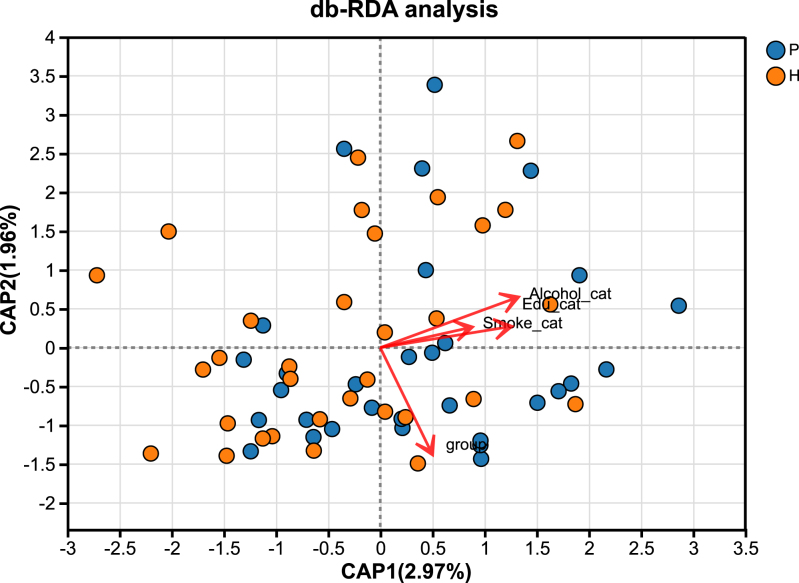
Distance-based redundancy analysis (db-RDA) of salivary microbiome variation in relation to group, education level, smoking history, and alcohol intake based on their original categorical coding. Arrows indicate the direction of association of the categorical factors with microbiome variation. This analysis was performed as a supplementary exploratory assessment and was not intended as a formal covariate-adjusted model.

We next examined whether patterns observed in the serum metabolome were accompanied by differences in the oral microbiome. Measures of alpha diversity, including Chao1 and Shannon indices, were comparable between the PND and HC groups (Fig. 3(a)) (Callahan et al., 2016). Consistently, beta-diversity analysis based on Bray–Curtis dissimilarity showed substantial overlap between groups, with no clear separation observed at the global community level (Fig. 3(b)) (Shi et al., 2025). Supplementary db-RDA based on the original categorical coding of education level, smoking history, and alcohol intake showed that all three variables were significantly associated with microbiome variation (Supplementary Figure S1 and Table S1).

Taxonomic profiling identified modest compositional differences between groups. In particular, a higher relative abundance of the phylum Proteobacteria and selected genera, including *Abiotrophia* and *Neisseria*, was observed in the PND group (Fig. 3(d)–3(e)) (Malan-Müller et al., 2024). Despite these differences, the overall taxonomic structure of the oral microbial community remained broadly similar between PND and HC participants.

In contrast to the largely preserved community structure, differences were observed at the level of inferred functional profiles derived from shotgun metagenomic annotation. Functional pathways related to lipopolysaccharide biosynthesis, bacterial secretion systems, and microbial energy metabolism showed higher relative representation in the PND group (Fig. 3(c)) (Franzosa et al., 2018). Analysis of virulence-associated functional categories based on the Virulence Factor Database further indicated differences in aggregated functional groups, including categories related to type IV pili, hemolysins, and other virulence-associated features, in PND samples (Fig. 3(f)) (Liu et al., 2019).

### 3.4 Limited global concordance but reproducible pathway-level cross-omic correspondence

**Fig. 4:**
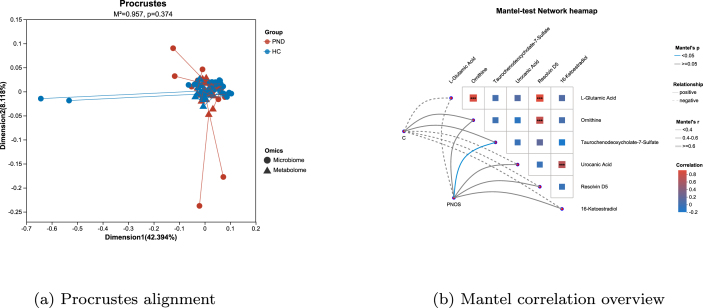
Cross-omic integration of the salivary metagenome and serum metabolome in women with perinatal depression (PND) and healthy controls (HC). (a) Procrustes analysis showed limited global concordance between microbial functional profiles and serum metabolomic profiles. (b) Mantel analysis identified selected pathway- and feature-level associations despite the limited global alignment.

To examine relationships between the oral microbiome and the serum metabolome, correspondence was evaluated at both global and pathway levels. Procrustes analysis based on distance matrices did not identify significant global alignment between salivary metagenomic functional profiles and serum metabolomic profiles ($M^{2}=0.957$, p=0.374; Fig. 4a) (Paliy and Shankar, 2016), indicating limited concordance at the overall data structure level.

**Fig. 5:**
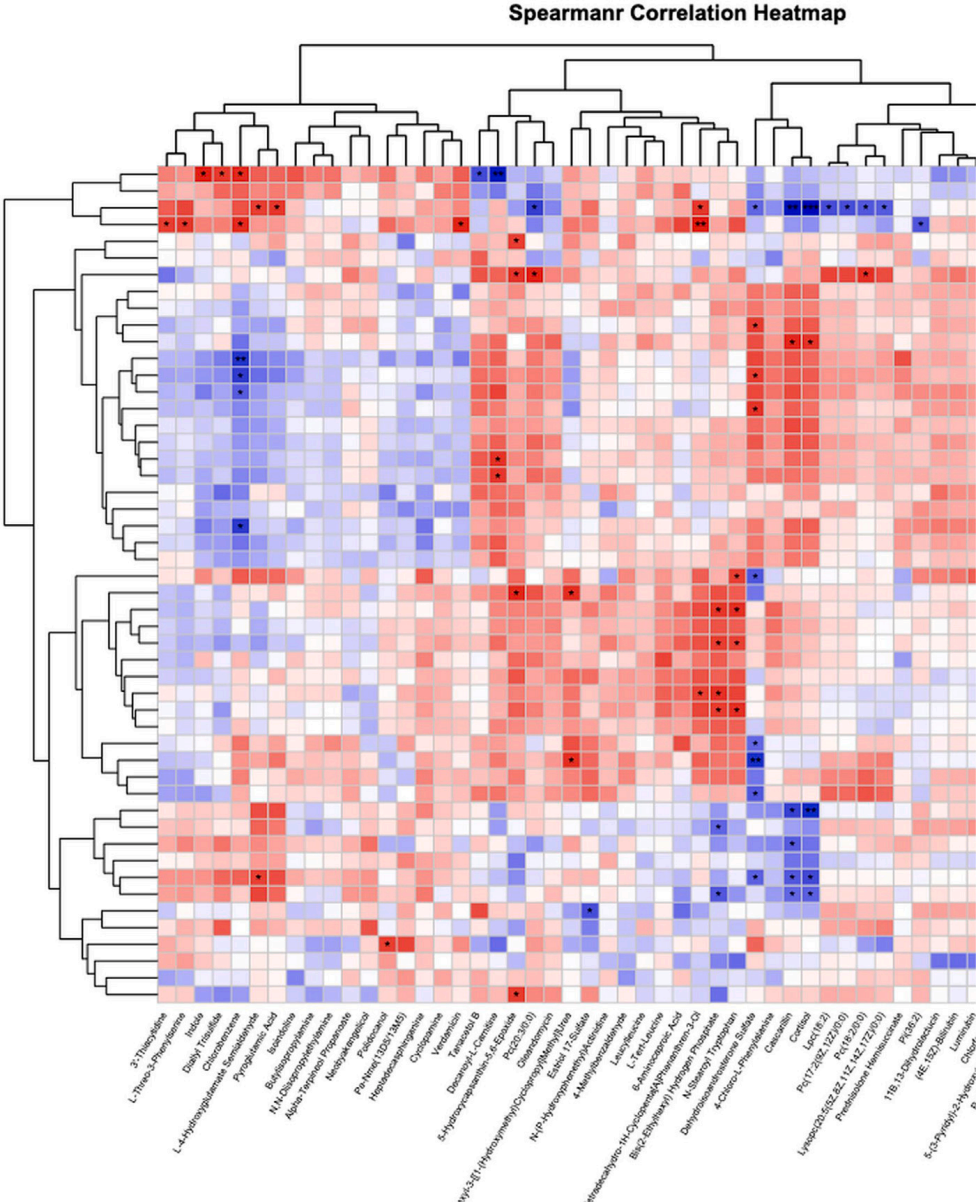
Cross-omic correlation heatmap of selected salivary taxa and serum metabolites in women with perinatal depression (PND) and healthy controls (HC). The heatmap highlights a subset of significant positive and negative associations after multiple-testing correction, indicating selective cross-omic relationships rather than broad global concordance.

**Fig. 6:**
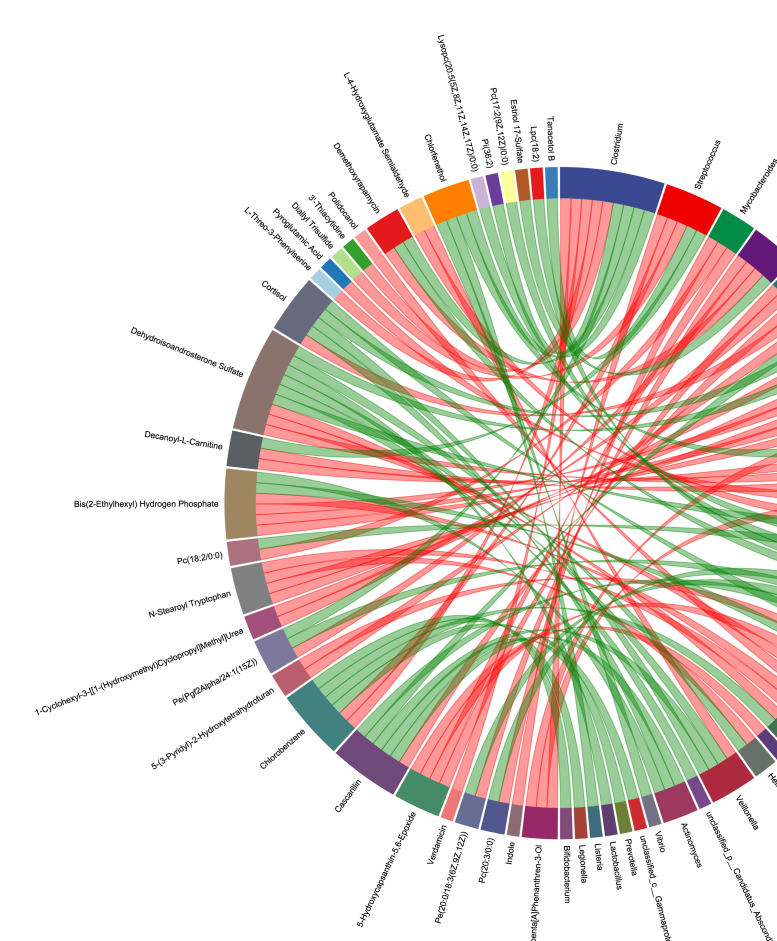
Chord diagram of significant cross-omic associations between selected salivary taxa and serum metabolites. Red and green links indicate positive and negative associations, respectively. The diagram highlights a subset of significant taxon-metabolite pairs, consistent with selective cross-omic relationships rather than broad global concordance.

At finer analytical resolution, selective correspondence was observed. Mantel tests identified associations between subsets of microbial functional features and groups of serum metabolites, with correlation coefficients in the low-to-moderate range (Fig. 4b). Additional feature-level correlation analyses highlighted reproducible associations between selected oral taxa or functional categories and individual serum metabolites. As an illustrative example, the relative abundance of the genus *Abiotrophia* was inversely associated with levels of *taurochenodeoxycholate-7-sulfate* (Figs. 5 and 6) (Huang et al., 2024).

Together, these results describe a pattern in which global correspondence between omics layers is limited, while selective associations can be detected at the pathway or feature level.

### 3.5 Integrated feature sets show moderate internal separability

To examine whether the identified molecular features contained group-related discriminative information, supervised classification analyses were performed using selected serum metabolites and oral microbial features. A compact serum-based feature set, including *taurochenodeoxycholate-7-sulfate*, *urocanic acid*, and putatively annotated *resolvin D5*, showed moderate internal separability between PND and HC groups under nested cross-validation, with an area under the receiver operating characteristic curve of approximately 0.77 (Fig. 2d) (Tejavibulya et al., 2022).

The inclusion of selected microbial features resulted in only minor changes in classification performance. Overall, these analyses indicate that the identified molecular features contain limited to moderate internal group-related separability. This analysis was included as a descriptive assessment of feature informativeness and was not intended to develop or evaluate a predictive or diagnostic model.

## 4 Discussion

### 4.1 Systemic but asynchronous alterations in perinatal depression

In this study, integrated analysis of salivary metagenomic and serum metabolomic data offers a systems-oriented description of biological variation observed in women with perinatal depression (PND). Rather than pointing to a single localized alteration, the results are consistent with deviations from typical perinatal physiological patterns across multiple biological compartments.

Across compartments, the data describe a systemic but non-uniform pattern of variation. The serum metabolome exhibited coordinated differences at the level of endocrine- and immune-related metabolic pathways, whereas the oral microbiome showed differences mainly in inferred functional profiles despite largely preserved community structure. Global concordance between these two molecular layers was limited, while selective correspondence was observed at the pathway level.

Viewed together, these findings suggest that biological changes associated with PND are not fully aligned across physiological systems. Instead, different biological compartments may vary partly independently during the perinatal period (Su et al., 2022, Xie et al., 2024). The limited global concordance between oral microbial functional profiles and systemic metabolic patterns should therefore be interpreted as an empirical finding rather than a limitation of the analysis.

From a translational perspective, these findings suggest that different biological compartments may capture different aspects of heterogeneity in perinatal depression. Oral and systemic molecular profiles may therefore provide complementary information when characterizing system-level variation during the perinatal period.

### 4.2 Functional alterations of the oral microbiome during the perinatal period

Within the oral compartment, the present results describe a dissociation between microbial community structure and inferred functional profiles. Standard measures of alpha and beta diversity showed minimal differences between women with PND and healthy controls, indicating that large-scale taxonomic disruption was not a prominent feature of this cohort.

In contrast to the largely preserved global community structure, modest taxonomic differences were observed at the phylum, genus, and species levels, while clearer differences were detected in inferred functional pathway representation. Functional categories related to lipopolysaccharide biosynthesis, bacterial secretion systems, and virulence-associated functions showed higher relative representation in the PND group. Together, these findings suggest that oral microbial functional variation may be more apparent than taxonomic variation in this cohort. Importantly, these annotations reflect inferred functional potential based on gene content and do not imply active pathogenicity, infection, or ongoing host inflammatory responses (Cobb et al., 2017).

In the context of the perinatal period, which involves substantial hormonal and immune adaptation, these functional differences may reflect shifts in microbial functional orientation rather than acute pathological processes. Rather than indicating infection or structural instability, these patterns point to differences in functional features that may be relevant to host–microbe interactions at barrier surfaces. Together, these findings emphasize the value of functional profiling alongside taxonomic characterization when examining oral microbial features in relation to perinatal mental health (Hashimoto, 2023).

### 4.3 Systemic metabolic alterations reflect changes in host regulatory tone

In parallel with observations in the oral microbiome, serum metabolomic profiles in women with PND showed coordinated differences across multiple pathways related to endocrine- and immune-associated processes. Group-associated metabolite features were enriched in pathways involving bile acid metabolism, steroid-related metabolites, amino acid and redox metabolism, and lipid mediator-associated classes (Wishart et al., 2018, Thomas et al., 2008). These differences were not limited to metabolites commonly linked to inflammatory activation, but also involved metabolic classes associated with regulation and resolution, including bile acids with immunomodulatory associations and putatively annotated lipid mediators such as resolvin D5 (Fullerton and Gilroy, 2016, Luo et al., 2019).

These metabolomic patterns do not necessarily indicate a generalized increase in inflammatory burden. Instead, they suggest differences in metabolic pathways involved in immune-metabolic regulation during the perinatal period. Variation in bile acid composition and lipid mediator-associated features may reflect broader shifts in host metabolic and endocrine context during pregnancy and the postpartum transition (Zeng et al., 2017). Taken together, the serum metabolomic findings are more consistent with altered immune-metabolic balance than with isolated inflammatory activation.

### 4.4 Limited global concordance and selective cross-omic associations

Integrating oral microbial and serum metabolic data offers a way to examine how compartment-specific patterns of variation may relate across biological systems. In the present study, global alignment between microbial functional profiles and circulating metabolic features was limited, while reproducible correspondence was observed at the level of selected pathways and features. This combination of limited overall concordance and selective local associations is consistent with non-uniform organization across biological compartments in PND (Rosario et al., 2024).

These findings indicate that oral microbial functional categories related to lipopolysaccharide biosynthesis and virulence-associated functions varied alongside serum metabolic features including bile acids and putatively annotated lipid mediators such as resolvin D5 (Sedghi et al., 2021). However, this co-occurrence does not imply direct functional coupling or impaired host regulation. Instead, it suggests that microbial functional features and host metabolic pathways may vary partly independently during the perinatal period. The observed combination of limited global concordance and selective pathway-level associations should therefore be interpreted without assuming direct causality or synchronized regulation between microbial and host systems.

### 4.5 Functional variation beyond taxonomic stability

An important observation in this cohort is that functionally annotated features were not fully captured by conventional taxonomic metrics. Although the oral microbial community showed largely stable taxonomic organization, differences in inferred functional pathways were still detectable, indicating that compositional measures alone may miss relevant dimensions of microbial variation (Clemente et al., 2018).

Such functional features may be observed even in the absence of overt dysbiosis or clinically evident inflammatory markers, and they should not be interpreted as evidence of a distinct pathological state. Instead, they reflect differences in functional orientation inferred from gene content and annotation. This may be particularly relevant during the perinatal period, when subtle system-level variation can occur across multiple physiological systems, and it supports the value of combining functional characterization with taxonomic profiling in studies of perinatal mental health (Sedghi et al., 2021, Safadi et al., 2022).

### 4.6 Limitations and future directions

Several limitations should be considered when interpreting the findings of this study. The cross-sectional design does not allow assessment of temporal ordering or causal relationships among oral microbial functional profiles, systemic metabolic variation, and depressive symptoms. Longitudinal studies spanning pregnancy and the postpartum period will therefore be required to determine whether the observed cross-omic patterns precede, coincide with, or follow the development of perinatal depressive symptoms (Rahnavard et al., 2024).

In addition, participants were sampled across late pregnancy and the early postpartum period, a phase characterized by rapid endocrine and immunological adaptation. Although the distribution of sampling periods was balanced between groups, residual heterogeneity related to hormonal dynamics, delivery-associated exposures, and postpartum physiological adjustment cannot be fully excluded and may contribute to variability in metabolic profiles.

The relatively modest sample size, particularly for serum metabolomic analyses, also limits generalizability and highlights the need for replication in larger, multi-center cohorts (Zhou et al., 2024). Education level, smoking history, and alcohol intake were collected as categorical self-report variables with partial missingness and were not modeled as continuous covariates. Residual confounding related to these lifestyle and socioeconomic factors therefore cannot be fully excluded. Moreover, metagenomic functional profiles are inherently compositional, and correlation-based analyses using relative abundances may be influenced by spurious associations. To mitigate this, the present analyses emphasized pathway-level patterns and cross-omic consistency rather than isolated feature-level relationships.

Interpretation of specific functional and metabolic features should be approached with caution. VFDB-based annotations reflect variation in virulence-associated functional potential encoded in microbial genomes and do not indicate active pathogenicity or infection. Similarly, owing to the untargeted nature of the metabolomics platform, individual metabolite identities represent putative annotations rather than definitive molecular assignments. Accordingly, emphasis was placed on coordinated patterns at the pathway and lipid-class levels rather than on attribution to individual molecular species.

Finally, this study did not include direct measurements of circulating cytokines, immune cell populations, or dynamic endocrine markers. Future work integrating longitudinal multi-omics with immunological and endocrine profiling will be important for clarifying how microbial functional features and host metabolic pathways related to immune regulation evolve over time. Such designs may also help to further assess the relevance of oral and systemic molecular patterns for understanding biological heterogeneity in perinatal mental health (Fasano et al., 2024).

## 5 Conclusion

This study integrates salivary metagenomic and serum metabolomic profiling to characterize oral microbial functional features and systemic metabolic patterns in women with perinatal depression. The results indicate that, despite largely preserved microbial community structure and limited global cross-omic concordance, differences can be detected at the level of inferred functional pathways and metabolic classes.

The observed pattern of limited global alignment combined with selective pathway-level correspondence suggests that biological variation associated with perinatal depression may be organized across multiple physiological compartments, rather than manifesting as a single coordinated molecular signature. From this perspective, oral microbial functional profiles and systemic metabolic features may reflect different aspects of immune–metabolic variation during the perinatal period.

By emphasizing functional characterization within a multi-omics framework, this work contributes to a more nuanced, systems-level description of biological heterogeneity associated with perinatal depression and provides a foundation for future longitudinal studies aimed at clarifying temporal dynamics across biological compartments.

## CRediT authorship contribution statement

**Kexin Xue:** Writing – original draft, Methodology, Investigation, Formal analysis, Conceptualization. **Chunfei Hu:** Writing – original draft, Visualization, Data curation. **Zhehao Lin:** Resources, Investigation. **Xiaokai Mao:** Validation, Software. **Haihua Zhu:** Resources. **Yanyi Xie:** Investigation. **Qiong Luo:** Writing – review & editing, Supervision, Resources, Conceptualization. **Fudong Zhu:** Writing – review & editing, Supervision, Resources, Project administration.

## Declaration of generative AI and AI-assisted technologies in the manuscript preparation process

During the preparation of this work, the author(s) used ChatGPT (OpenAI) to assist in grammar and language polishing. The author(s) reviewed and verified all outputs and take full responsibility for the final content.

## Funding

This work was supported by National Key Research and Development Program of China (grant number 2022YFC2402903); the “Pioneer” and “Leading Goose” R&D Program of Zhejiang (grant number 2025C02102); the 10.13039/501100001809National Natural Science Foundation of China, Professor Fudong Zhu’s Project (grant number 82471034); and the Provincial Natural Science Foundation of Zhejiang , Professor Yan Yi Xie’s Project (grant number LTGY23H140006).

## Declaration of competing interest

The authors declare that they have no known competing financial interests or personal relationships that could have appeared to influence the work reported in this paper.

## Figures and Tables

**Table C.2:** Continuous variables summary.

Variable	Overall	HC	PND
Age (years)	30.0 ± 4.6 (n = 63)	30.7 ± 3.5 (n = 32)	29.4 ± 5.5 (n = 31)
Pre-pregnancy BMI (kg/m${}^{2}$)	22.0 ± 4.3 (n = 63)	21.6 ± 2.4 (n = 32)	22.5 ± 5.7 (n = 31)
Gestational age at enrollment (weeks)	38.5 ± 1.3 (n = 63)	38.7 ± 1.1 (n = 32)	38.3 ± 1.5 (n = 31)
Gestational age at delivery (weeks)	38.9 ± 1.2 (n = 63)	39.0 ± 1.1 (n = 32)	38.7 ± 1.4 (n = 31)
Birth weight (g)	3350.0 ± 316.7 (n = 63)	3368.4 ± 266.0 (n = 32)	3331.0 ± 365.3 (n = 31)
PHQ-9 score	8.0 ± 7.3 (n = 63)	1.3 ± 1.9 (n = 32)	14.9 ± 2.9 (n = 31)

**Fig. S2:**
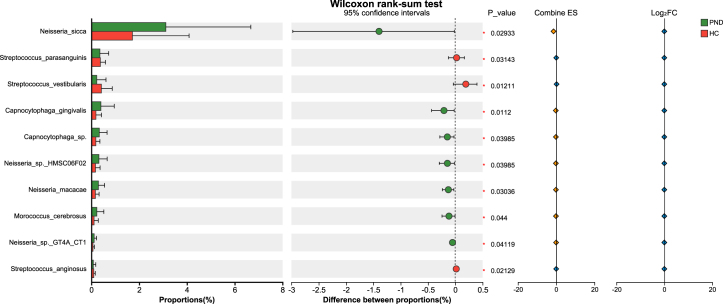
Species-level differential abundance between PND and HC groups. Bar plots show the relative abundance of key species that significantly differ between PND and HC groups based on the Wilcoxon rank-sum test (95% confidence intervals are shown). Green bars represent the PND group and red bars represent the HC group. The right panels display the corresponding P-values, combined effect sizes, and log fold changes. Differential species such as Neisseria sicca and Streptococcus parasanguinis follow the same trends observed at the genus level, confirming consistent cross-level patterns in community composition.

**Fig. S3:**
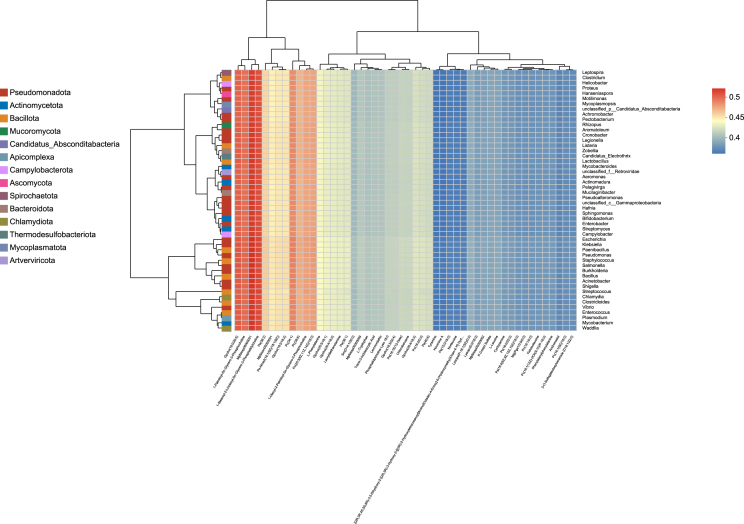
MMvec-derived microbe–metabolite co-occurrence heatmap. This heatmap displays the co-occurrence probabilities between major microbial taxa and serum metabolites estimated by MMvec modeling. Each cell represents the conditional probability of observing a metabolite given the presence of a specific taxon. Higher values indicate stronger co-occurrence, which suggests potential metabolic interactions or shared ecological niches. The heatmap highlights taxa–metabolite pairs enriched for amino acid and lipid metabolism pathways, supporting the cross-omics relationships shown in the main figure.

**Table S1:** Db-RDA environmental factor statistics for group, education level, smoking history, and alcohol intake.

Variable	CAP1	CAP2	$R^{2}$	P value
group	0.4929	−0.8701	0.2858	0.001
Edu_cat	0.9999	0.0101	0.1982	0.004
Smoke_cat	0.9960	0.0892	0.0946	0.045
Alcohol_cat	0.9621	0.2728	0.2378	0.002

## Data Availability

Data will be made available on request.

## Appendix A Cohort and sampling

This study includes 63 mother participants. Group allocation shows 31 in PND and 32 in HC (49.2% and 50.8%). Saliva sampling covers 44 prenatal and 19 postpartum visits (69.8% and 30.2%). Clinical confirmation of PND is available in 5 records.

Sampling distribution by group is balanced. HC shows 23 prenatal and 9 postpartum visits. PND shows 21 prenatal and 10 postpartum visits.

Delivery mode distribution shows 33 cesarean sections, 29 vaginal deliveries, and 1 forceps-assisted delivery (52.4%, 46.0%, and 1.6%).

## Appendix B Coding of key variables

• Group: 1 PND, 2 HC.
• EPDS score: integer score. The range in this dataset is 0 to 30.
• Clinical PND diagnosis: 1 yes, 0 no.
• Sampling period for saliva: 1 prenatal, 2 postpartum.
• Delivery mode: vaginal, cesarean, or forceps-assisted.
• Anthropometrics and timing: age in years, height in cm, current weight in kg, gestational week at enrollment, gestational week at delivery, pre-pregnancy BMI in kg/m^2^.
• Lifestyle and support (self-report): smoking history (never, <100 cigarettes lifetime, ≥100 cigarettes lifetime); alcohol intake (never, occasional, weekly or more); perceived family support during pregnancy and postpartum (low, occasional, mostly, consistent).

## Appendix C Descriptive statistics

Continuous variables are summarized as mean ± SD with sample size in parentheses (see Table C.2).

## Appendix D Selected cohort characteristics

**Sampling and diagnosis**

• PND vs HC: 31 vs 32 (49.2% vs 50.8%).
• Prenatal vs postpartum sampling: 44 vs 19 (69.8% vs 30.2%).
• Clinical PND diagnosis available in 5 participants.

**Delivery mode**

• Cesarean section 33 (52.4%), vaginal delivery 29 (46.0%), forceps-assisted delivery 1 (1.6%).

**Lifestyle and support**

• Smoking history: never 42 (66.7%), <100 cigarettes 8 (12.7%), ≥100 cigarettes 6 (9.5%); missing 7 (11.1%).
• Alcohol intake: never 35 (55.6%), occasional 21 (33.3%); missing 7 (11.1%).
• Perceived family support: consistent 24 (38.1%), mostly 23 (36.5%), occasional 9 (14.3%); missing 7 (11.1%).

## Appendix E Pregnancy comorbidities snapshot

Text entries were standardized at the term level. The most frequent items include gestational diabetes 13, polyhydramnios 4, hypothyroidism after thyroid surgery 4, obesity 3, premature rupture of membranes 3, subclinical hypothyroidism 2, chronic hypertension with severe preeclampsia 2.

Missingness for lifestyle and support is 7 of 63. Socioeconomic items such as education, job status, and household income are present with partial missingness. These items are not used in the primary models in this manuscript.

## Supplementary figures

See Fig. S1, Fig. S2, Fig. S3.

## Supplementary tables

See Table S1.
